# The Association of Short-Chain Fatty Acids with the Occurrence of Gastrointestinal Symptoms in Infants

**DOI:** 10.3390/ijms252312487

**Published:** 2024-11-21

**Authors:** Małgorzata Szczuko, Gabriela Duliban, Arleta Drozd, Diana Sochaczewska, Kamila Pokorska-Niewiada, Maciej Ziętek

**Affiliations:** 1 Department of Human Nutrition and Metabolomics, Pomeranian Medical University in Szczecin, 71-460 Szczecin, Polandarleta.drozd@pum.edu.pl (A.D.); 2 Department of Human Nutrition and Bromatology, Pomeranian Medical University in Szczecin, 71-210 Szczecin, Poland; 3 Department of Neonatology, Pomeranian Medical University in Szczecin, 72-009 Police, Poland; diana.sochaczewska@pum.edu.pl; 4 Department of Toxicology, Dairy Technology and Food Storage, West Pomeranian University of Technology in Szczecin, 71-459 Szczecin, Poland; kamila.pokorska@zut.edu.pl; 5 Department of Perinatology, Obstetrics and Gynecology Pomeranian Medical University in Szczecin, 72-009 Police, Poland; maciej.zietek@pum.edu.pl

**Keywords:** SCFA, microbiota, infants, gastrointestinal disorders, gas, bloating, diarrhoea, constipation, intestinal colic, BSCFA, acetic, propionic, butyric, valeric acid

## Abstract

Short-chain fatty acids (SCFAs) are produced by the fermentation of undigested polysaccharides; they are a group of metabolites resulting from the activity of intestinal bacteria. The main SCFAs are acetic, butyric, propionic, valeric, and caproic acid, and their levels and proportions depend on various factors. The aim of this study was to investigate the relationship between the concentration of SCFAs and the occurrence of specific gastrointestinal symptoms in infants. This study was conducted using faecal samples obtained at 1, 3, 6, and 12 months of age. The SCFA content was measured using gas chromatography. At 1 month, an association was found between butyric acid and flatulence. At 3 months, an association was found between butyric acid and flatulence/gas and between 3,4-methylovaleric acid and mucus in the stool. At 6 months, an association was found between butyric and valeric acids and flatulence. By 12 months, the gastrointestinal symptoms had decreased significantly. This study confirms that there is an association between SCFA levels and the presence of bloating, gas, mucus in the stool, and constipation in the gastrointestinal tract. Higher levels of butyric and valeric acids may lead to an increase in troublesome symptoms, such as flatulence and gas, in the first few months of life but are not associated with the occurrence of intestinal colic. The level of 3,4-methylovaleric acid is associated with the presence of allergies, whereas a decrease in acetic acid and an increase in isovaleric acid may exacerbate defecation problems in infants.

## 1. Introduction

The most well-known short-chain fatty acids (SCFAs) include acetic acid, propionic acid, and butyric acid [1]. These acids are primarily produced in the colon by bacteria belonging to the families *Bifidobacterium, Bacteroides*, *Roseburia*, *Fecalibacterium*, and *Enterobacteria*. The content and mutual proportions of SCFAs depend on many factors. The type of diet and protein intake are two of the most important factors affecting the development of the gut microbiome and the presence of SCFAs. Other factors related to the concentration of SCFAs include human genotype and physiology, age, health status or diagnosed diseases, physical activity, chronic stress, lifestyle, and medication use. The internal factors that regulate the concentration of SCFAs in the intestines include the composition of the gut microbiota, metabolism, peristalsis, and intestinal transit [2,3]. SCFAs are metabolites produced by the fermentation of dietary fibre by the gut microbiota [3]. The resulting SCFAs are responsible for the proper functioning of the intestinal epithelium and the intestinal barrier. As the human diet has changed considerably, with a decrease in the intake of soluble and insoluble fibre from plant-based products compared to previous generations, there have also been changes in the proportions and amounts of SCFAs synthesised [4]. This trend is particularly noticeable in highly developed countries, where the population is more likely to face autoimmune diseases [5]. It is suspected that SCFAs play an important role in signalling and activating the immune system.

Maternal microbiota has been shown to influence antibody production, including IgA, IgM, and IgG, in their offspring [6]. When breastfed, infants show greater diversity in the types of bacteria present, including *Bifidobacterium*, *Lactobacillus* spp., *Streptococcus*, *Staphylococcus*, *Ralstonia*, *Bacteroides*, *Enterobacter*, and *Enterococcus* [7]. Following birth, infants develop their own distinctive gut microbiota, which is supported by breastfeeding. Further intestinal colonisation depends on contact with relatives, including the mother, as well as exposure to the bacteria present in the child’s environment. This process brings benefits to the infant, among them the acquisition of immunity, the management of microorganisms, and the reinforcement of the intestinal barrier. Undigested carbohydrates from the upper gastrointestinal tract are transported to the colon, where they are fermented by anaerobic microbiota [8]. In addition to their role as a source of energy, SCFAs serve signalling functions through receptors coupled with G proteins, as evidenced by studies [9,10]. This mechanism facilitates communication between the microbiota and the human immune system. It appears that the principal function of SCFAs is to regulate the equilibrium between anti-inflammatory and pro-inflammatory reactions. The SCFAs produced by the microbiota are present in the peripheral, hepatic, and portal blood and can be transported into the bloodstream or other organs. The liver and muscles are the primary organs responsible for the metabolism of SCFAs. Fatty acids exert an influence on glucose, cholesterol, and lipid metabolism in tissues. Once SCFAs are taken up by organs, they can perform the function of a signalling molecule or substrate [11].

### 1.1. Synthesis Pathways and Utilisation of SCFAs

The production of acetate by intestinal bacteria commences with pyruvate, subsequently forming acetyl-CoA or via the Wood–Ljungdahl pathway. Furthermore, acetate production in adults also occurs endogenously via ethanol oxidation [12]. The greatest concentrations of acetic acid are present in the peripheral circulation [13].

The second most commonly mentioned SCFA is propionate, which is formed via the succinate pathway, subsequent to the attachment of succinate to methylmalonyl-CoA. An alternative method of propionate production is through the synthesis of acrylates and lactates, which represents the initial stage of the acrylate pathway. Furthermore, it can be produced via the propanediol pathway, which consists of deoxyhexose sugars [12]. Lactic acid bacteria are also involved in the production of SCFAs. The fermentation of pyruvate in the glycolytic pathway or the phosphoketolase pathway is utilised by this bacteria [14]. The site of fatty acid metabolism is the colon, so propionate is found in low concentrations in the periphery. The investigators suggested that propionate, in addition to exhibiting anti-inflammatory properties, may play an important role in regulating appetite [15]. It has been demonstrated that propionate present in the colon assists in reducing energy intake and inhibits excessive weight gain in humans [16].

Butyric acid is the main energy source for colonocytes, providing 60–70% of their energy during metabolic processes, while other SCFAs are metabolised by the basolateral membrane. It has been demonstrated that butyric acid is crucial for maintaining proper colon function and plays a role in inflammatory processes and those caused by oxidative stress [17]. The presence of butyrate is essential for the proper functioning of the epithelium of the digestive tract and its normal development [18]. Additionally, it is worth noting that *Faecalibacterium prausnitzii* is one of the first commensal bacteria with anti-inflammatory properties to be identified in clinical studies in humans. The production of butyrate is also facilitated by bacteria, such as *Eubacterium*, *Butyrivibrio*, and *Clostridium*. During this process, intermediate products are formed. Butyrate is the final product of fermentation and can be used as an energy substrate, whereas propionate and acetate serve as energy substrates for peripheral tissues [19].

### 1.2. SCFA Signalling

Despite having fundamentally different functions, SCFAs’ synergistic effects on the intestinal mucosa contribute to maintaining structural integrity. SCFA receptors are expressed during host energy metabolism and adapt to changes in the gastrointestinal tract by stimulating the immune system and modulating signalling [20,21]. The free fatty acid receptors GPR43 and GPR41 help maintain balance in adipose tissue and the gastrointestinal tract. An interesting finding from studies on mice shows that those deficient in GPR43 became obese despite being fed a normal diet. Conversely, mice that were fed a high-fat diet with SCFAs and had an excess of GPR43 remained lean. GPR43 is an SCFA receptor activated by acetate, propionate, and butyrate [22]. It has been observed that the concentration of individual SCFAs varies depending on their exact location in the colon. Higher SCFA concentrations are noticeable in the proximal colon, while lower concentrations are found in the distal part [23]. The concentration of acetate throughout the colon is significantly higher and can be up to twice that of propionate or butyrate. Butyrate is mostly utilised by colonocytes, while acetate and propionate reach the liver. Due to saccharolytic fermentation, the levels of the main SCFAs differ, but acetate is the most prevalent in faeces [5,24].

It has been demonstrated that during the fermentation of complex carbohydrates, SCFAs play a significant role in protecting the development of the body’s immune system [25]. SCFAs regulate the production of pro-inflammatory cytokines in immune cells, a process that also occurs in endothelial cells. Acetate, propionate, and butyrate acids regulate the host’s immunity via increased IL-10 production and receptor-dependent repression of claudin-2 [26]. Their influence is becoming increasingly recognised in the context of metabolism [26].

### 1.3. Pregnancy and Breastfeeding

The following factors have been identified to modify the microbiome: the mother’s age and health status, the type of initial feeding, predispositions, genetics, environmental factors, and the mode of delivery, including the use of antibiotics [27,28]. The composition of the gut microbiota is altered by nutrition with formulas based on human milk, including the delayed introduction of enteral nutrition and the use of medications that inhibit gastric acid secretion. The combination of adverse environmental factors with the immature gastrointestinal and immune systems of infants results in the onset of clinical symptoms [29,30]. The immunological immaturity of newborns is associated with a poorly developed lymphatic system and an underdeveloped innate immunity [31]. Furthermore, an overly sterile environment impedes the acquisition of immunological competence.

It has been demonstrated that breastfeeding provides support to the immune systems of infants, induces favourable alterations in the structure of the intestinal microbiota, and ensures the optimal functioning of mucosal stimuli in the gastrointestinal tract [32,33]. This is related to the bioactive components of human milk, including antibodies, human milk oligosaccharides (HMOs), lactoferrin, immunoglobulins, hormones, and the milk microbiota itself [34]. Pregnancy itself gives rise to alterations in the composition of the gut microbiome of the expectant mother. The most notable differences are observed between the first and third trimesters of pregnancy. These changes include an increase in the number of bacteria belonging to the phyla *Actinobacteria* and *Proteobacteria*. The phylum *Proteobacteria* has been linked to inflammatory conditions. Furthermore, studies have shown that throughout the three trimesters of pregnancy, the number of *Bifidobacteria* and lactic acid-producing bacteria increases, while a decrease in individual bacterial diversity and a reduction in the number of bacteria responsible for butyrate production occur [32,33]. Human milk oligosaccharides (HMOs) are complex carbohydrates that serve a prebiotic function, supporting the colonisation of beneficial bacteria in the infant’s gastrointestinal tract. The content of HMOs is influenced by a number of factors, including the environment, genetics, and the timing of lactation [35].

The available literature has not yet analysed the influence of SCFAs on gastrointestinal symptoms in any age group. Therefore, we focus on pioneering studies in this area and the possibility of associations in infancy. Moreover, valeric acid is not as well researched as other SCFAs, and its presence in the gut is part of a healthy microbiome profile, which is why we included it in our research

## 2. Results

**Figure 1:**
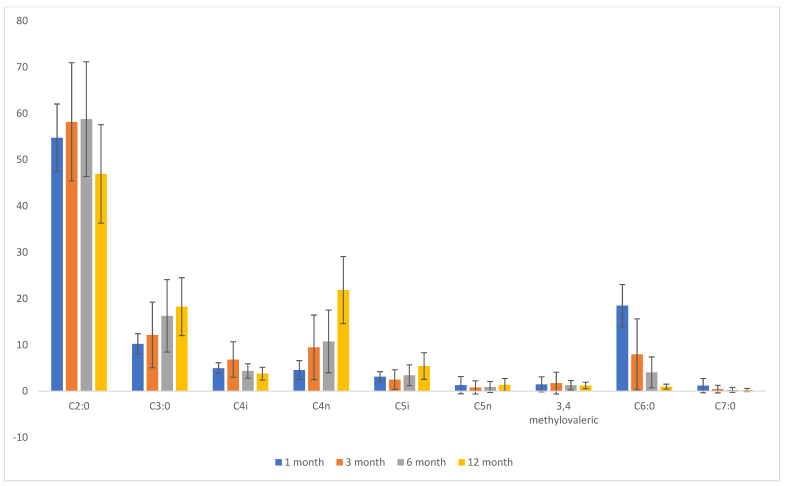
Mean SCFA levels at 1, 3, 6, and 12 months of infant life. C2:0—acetic acid; C3:0—propionic acid; C4i—isobutyric acid; C4n—butyric acid; C5i—isovaleric acid; C5n—valeric acid; C6:0—caproic acid; C7:0—heptanoic acid.

Table 1 presents the average contents of SCFAs at 1 month, 3 months, 6 months, and 12 months of the infants’ lives, as well as a comparison analysis of the means. The analysis includes the average contents of all the SCFAs studied: acetic acid, propionic acid, isobutyric acid, butyric acid, isovaleric acid, valeric acid, 3,4-methylvaleric acid, caproic acid, and heptanoic acid. It was observed that the average content of propionic, butyric, isovaleric, and valeric acids rose with the age of the children. In contrast, the average contents of caproic and heptanoic acids decreased with age (Table 1). The lowest standard deviations were recorded in the 1-month period, particularly for the average contents of fatty acids with shorter carbon chain lengths. Meanwhile, the standard deviations for 3,4-methylvaleric, caproic, and heptanoic acids decreased with age (Table 1). To enhance visibility, the results are also presented graphically (Figure 1). Based on the bar chart, it can be concluded that in the first month of the infants’ lives, the levels of fatty acids were lower, especially butyric acid, and the content of acetic acid stood out compared to all other SCFAs analysed in the first, third, sixth, and twelfth months of life (Figure 1).

Table 2 presents the correlations between the concentrations of SCFAs during the first year of life and the incidence of gastrointestinal symptoms. The symptoms evaluated include the presence of mucus in stool, bloating or flatulence, regurgitation, stool colour, stool consistency, and constipation.

In the initial month of life, a single correlation was identified between butyric acid and the presence of gas and bloating. No further significant correlations were identified between the remaining SCFAs and the gastrointestinal symptoms under evaluation.

By the third month, statistically significant correlations were observed between butyric and valeric acids and the presence of bloating and gas. Furthermore, the presence of mucus in the stool was correlated with 3,4-methylvaleric acid, which may indicate the activation of inflammatory processes and the immune system.

In the sixth month, correlations were identified between butyric, valeric, and 3,4-methylvaleric acids and the presence of bloating and gas. Additionally, a correlation was identified between constipation and reduced acetic acid levels and elevated 3,4-methylvaleric acid levels (Table 2).

In the twelfth month, a negative correlation was observed between 3,4-methylvaleric acid and the presence of mucus in stool, while a positive correlation was noted between isovaleric acid and the occurrence of constipation. No correlations were identified between the remaining SCFAs and gastrointestinal symptoms (Table 2).

In conclusion, this study identified a range of associations between SCFA levels and gastrointestinal symptoms in infants. The occurrence of both flatulence and bloating was documented at various stages of the infants’ development. The strongest correlation was observed between the occurrence of gas and bloating and butyric acid levels in infants aged 1, 3, and 6 months. The prevalence of gastrointestinal symptoms declined with age, with the twelfth month exhibiting the lowest incidence of symptoms among the patients, although the incidence of constipation increased, which was associated with higher levels of isovaleric acid.

## 3. Materials and Methods

This study was conducted on a cohort of newborns, with follow-up at three specific time points through to later stages of life. The objective of the study was to evaluate the correlation between SCFA levels and the prevalence of gastrointestinal symptoms in infants.

### 3.1. Characteristics of the Study Group

The study participants were women who had volunteered to take part in the project. The mean age of these women was 31.19 ± 4.52 years. These were singleton pregnancies that had been terminated at term. Most women had given birth for the first time (n = 41), 32 women had given birth for the second time, and 8 women were multiparous. The study cohort comprised 82 participants, with assessments conducted during routine paediatric examinations performed at 1, 3, 6, and 12 months of the infants’ lives. The majority of the infant group was female, representing 55% (n = 45) of the participants, while males constituted the remaining 45% (n = 37). Nearly half of the patients had a vaginal delivery (n = 35), and a caesarean section was performed in 47 women. Breastfed children made up the majority of the group (n = 55), with 17 supplemented children and 10 formula-fed children.

Stool samples were collected from the infants and stored in containers at a temperature of −80 °C until analysis. To ascertain the presence of gastrointestinal symptoms, a questionnaire was employed to evaluate the occurrence of mucus in the stool, bloating, excessive flatulence, intestinal colic, reflux, green stools, loose stools, and constipation. To determine the existence of digestive disorders, Rome IV diagnostic criteria were used [36]. Confirmation of the presence of intestinal dysfunction was considered when two of the seven characteristics shown were met. All symptoms were verified by one of the child’s caregivers and a paediatrician responsible for collecting the stool samples. The characteristics of the study group are presented in Table 3.

### 3.2. Measurement of SCFAs

A quantity of 0.5 g of each stool sample was suspended in a test tube containing 5 mL of distilled water. The tubes were agitated for a period of five minutes using a shaker. Subsequently, the pH of the suspension was adjusted to a range of 2–3 using a 5 M HCl solution [37]. The samples were then subjected to a 10 min shaking and 20 min centrifugation process at 5000 rpm. The resulting supernatant was filtered through a filter with an aperture of 400 µm·min and transferred to chromatographic vials for analysis via gas chromatography. The following SCFAs were analysed using an Agilent Technologies 1260 A GC system with a flame ionisation detector (FID): acetic acid (C2:0), propionic acid (C3:0), isobutyric acid (C4i), butyric acid (C4n), isovaleric acid (C5i0), valeric acid (C5n), 3,4-methylvaleric acid, caproic acid (C6:0), and heptanoic acid (C7:0). A capillary column of fused silica with a free fatty acid phase (DB-FFAP, 30 m × 0.53 mm × 0.5 µm) was employed [8,38]. The carrier gas was hydrogen, with a flow rate of 14.4 millilitres per minute. The heating process commenced at 100 °C for a period of 0.5 min, after which the temperature was increased to 180 °C at a rate of 8 °C per minute. The subsequent step involved an increase in temperature to 200 °C. The temperature was maintained for a period of five minutes. A volume of 1 µL of the sample extract was utilised, with the analysis of a single sample requiring a total of 17.5 min. Fatty acid identification was based on a comparison of the retention times of the analysed acids with those of commercially available standards [8,38].

### 3.3. Statistical Analysis

Statistical analysis was conducted using Statistica 13.3 software (Statsoft, Kraków, Poland). The Shapiro–Wilk parametric test was employed, with a significance level of *p* ≤ 0.05.

## 4. Discussion

The formation of SCFAs is a consequence of carbohydrate fermentation in the large intestine. The fermentation of polysaccharides and oligosaccharides is primarily conducted in the proximal colon by saccharolytic bacteria, resulting in the linear production of n-SCFA, H_2_, and CO_2_. In contrast, the fermentation of amino acids or proteins is associated with the production of branched-chain SCFAs (BSCFAs), H_2_, CO_2_, CH_4_, phenols, and amines, primarily from valine, leucine, and isoleucine [39]. This fermentation is mainly conducted by *Bacteroides* and *Clostridium species*. All SCFAs regulate mucus production, intestinal pH, and epithelial cell nutrition and protect mucosal membranes [40]. They are involved in metabolic processes (regulation of glucose balance) that are essential for maintaining optimal gut function. For example, an increase in propionic acid levels has been observed to stimulate insulin secretion and activate the GLP-1 peptide, which has been demonstrated to have protective effects against cytokine-induced programmed cell death [41]. Propionate binds to free fatty acid receptors (FFAR2 and FFAR3) and activates insulin in pancreatic islets, dependent on protein kinase C activation [42,43]. Acetate and butyrate represent the primary substrates for lipogenesis. Furthermore, they serve as substrates in the formation of cholesterol. In contrast, propionate functions in an opposing manner. It has been demonstrated that the oral administration of propionate results in alterations in the levels of specific enzymes in the serum, including aspartate aminotransferase (AST), alanine aminotransferase (ALT), and alkaline phosphatase (ALP), which consequently lead to liver damage [43]. Conversely, butyric acid has been demonstrated to inhibit the expressions of cytokines IL-6, IL-1β, and TNFα, thereby exhibiting anti-inflammatory effects [44].

Published studies have demonstrated that the quantity of SCFAs and intestinal gases increases with age in children during the initial four weeks of life [45]. Our findings demonstrate that the production of SCFA increases with age in infants, with propionic, butyric, isovaleric, and valeric acids being produced in greater quantities. Furthermore, we observed significant increases in these acids between the third and sixth months of life, which supports the hypothesis that the colonisation of the infant gastrointestinal tract by the microbiome plays a role in these changes. Moreover, the significant discrepancies in standard deviations observed in older infants can be attributed to the influence of environmental factors that differ from those experienced during pregnancy. It can, therefore, be concluded that the method of feeding and delivery, as well as contact with food, represents a significant differentiating factor, a conclusion that is supported by other studies [46]. One of the most common symptoms of the digestive system in children, particularly during the early stages of life, is infantile colic. The aetiology of intestinal colic remains unknown. Nevertheless, it is postulated that intestinal colic (IC) is contingent upon the interplay of multiple factors, encompassing gastrointestinal disorders, neurodevelopmental, psychosocial, and hormonal factors. The literature indicates that the prevalence of these disorders ranges from 8 to 40%.

This condition is characterised by prolonged crying and screaming for no apparent reason [47,48]. In the 2021–2022 period, research was conducted in Kazakhstan on newborns and older children with IC. The studies demonstrated that the microbiome of infants with IC was markedly distinct from that of children without IC [44]. The inability to establish a normal intestinal flora may result in increased gas production or advanced inflammation, both of which are frequently associated with IC crying. It was demonstrated that in children with increased intestinal gas production, which is caused by the presence of *E. coli* and *Klebsiella*, there is a reduced presence of anti-inflammatory bacteria, such as *Bifidobacterium* and *Lactobacillus* [47]. This was largely influenced by the microorganisms that were transferred during the process of delivery. The patients with IC exhibited a higher microbial content, which included the following: The presence of *Veillonella ratti*, *Roseburia*, and *Anaerobutyricum hallii* resulted in the formation of gas within the intestines. An increased production of intestinal gases, including hydrogen, methane, and carbon dioxide, can cause discomfort and contribute to the occurrence of IC [41]. It has been demonstrated that the risk of developing a functional gastrointestinal disorder or IC is significantly higher in infants who are formula-fed compared to those who are breastfed. In this study, it was observed that a thickened milk formula with starch and reduced lactose, containing Limosilactobacillus reuteri DSM 17938 and a mixture of fructooligosaccharides and galactooligosaccharides, was effective in alleviating the symptoms of IC and regurgitation. However, further research is required to confirm these findings [48]. A number of factors may contribute to the occurrence of IC in infants. Further research is required to elucidate the relationship between the intestinal microbiome, which is complex and dynamic, and infant IC [49]. The results of our own study do not support the supposed correlation between the occurrence of IC and the concentration of SCFAs (acetic, propionic, butyric, valeric, caproic, and heptanoic acid). This finding is seemingly intuitive, given the considerable distance between the site of IC and the location of SCFA synthesis. However, an increase in butyric and valeric acid during breastfeeding and formula feeding up to six months was observed to be associated with an occurrence of flatulence and gases. A number of studies have been conducted to determine the relationship between the occurrence of infantile IC and the consumption of milk and formula-fed infants. The consumption of cow’s milk in infant formulas may be a primary contributor to the occurrence of infant IC [50]. Following the introduction of dietary supplementation (vegetable soups) at six months, these relationships were no longer observed, which may be related to the soluble fibre content. It is noteworthy that a positive correlation was observed between 3,4 methyl and the occurrence of mucus in the intestines during the period of 3 to 6 months. This may indicate that this compound stimulates the immune system. Conversely, a negative correlation was observed during the period of 12 months of life. This may be attributed to the lack of stabilisation in the synthesis process. The prevalence of constipation among children represents a significant and pervasive global health concern. The health issue predominantly affects children born in Europe and America, with an estimated prevalence of 9.5%. A lower frequency was observed in Asian children. It is likely that this is related to dietary habits and socio-cultural factors. The primary method for diagnosing this condition is through clinical assessment, which was conducted in accordance with the Rome IV criteria [36]. This scale was selected due to the unavailability of a more suitable scale for younger children. Our findings indicate that the oldest children exhibited the highest prevalence of constipation. This was associated with a lower concentration of acetic acid in the 6th month and a higher concentration of isovaleric acid in the 12th month.

## 5. Conclusions

The quantity of SCFAs increases gradually in infants up to 12 months of age, while the levels of caproic and heptanoic acids decrease. Both of these acids were unrelated to the infant symptoms studied. SCFAs have been linked to gastrointestinal symptoms in infants, including bloating, flatulence, mucus, and constipation. Nevertheless, no direct association was observed between SCFA levels and the occurrence of intestinal colic, regurgitation, green stools, or loose stools. The presence of butyric and valeric acids has been linked to an increased prevalence of bloating and gas in infants during the early months of life. This effect has been observed to diminish following the introduction of a more varied diet. Conversely, an increase in 3,4-methylvaleric acid was observed to result in the appearance of mucus in infant stools, which is indicative of an inappropriate immune response and the potential for allergic reactions. The levels and proportions of SCFAs have the potential to serve as biomarkers for the assessment of gut function. However, further research is required in a larger population, taking into account different feeding methods, in order to confirm this hypothesis.

## Figures and Tables

**Table 1:** Average SCFA content by month of infant life.

	Mean ± SD								
Months	C2:0	C3:0	C4i	C4n	C5i	C5n	3,4-Methylovaleric	C6:0	C7:0
1	54.74 ± 6.28	10.22 ± 2.19	4.96 ± 1.17	4.56 ± 2.04	3.12 ± 1.08	1.29 ± 1.87	1.45 ± 1.63	18.48 ± 4.57	1.19 ± 1.54
3	58.16 ± 12.79	12.15 ± 7.09	6.82 ± 3.84	9.46 ± 6.97	2.49 ± 2.13	0.79 ± 1.43	1.72 ± 2.37	7.96 ± 7.66	0.43 ± 0.82
6	58.75 ± 12.40	16.27 ± 7.84	4.35 ± 1.57	10.74 ± 6.77	3.42 ± 2.26	0.89 ± 1.19	1.28 ± 1.02	4.06 ± 3.33	0.25 ± 0.53
12	46.90 ± 10.64	18.26 ± 6.26	3.77 ± 1.38	21.83 ± 7.24	5.42 ± 2.88	1.38 ± 1.35	1.22 ± 0.70	0.97 ± 0.53	0.24 ± 0.35
Correlation	C2:0 *p*-value	C3:0 *p*-value	C4i *p*-value	C4n *p*-value	C5i *p*-value	C5n *p*-value	3,4-Methylovaleric *p*-value	C6:0 *p*-value	C7:0 *p*-value
1 vs. 3	0.0002	0.000000	0.000000	0.000000	0.000001	0.0242	0.0039	0.0001	0.000000
1 vs. 6	0.0001	0.000000	0.0237	0.000000	0.000000	0.000000	0.0001	0.0103	0.000000
1 vs. 12	0.0088	0.000000	0.2370	0.000000	0.000000	0.0256	0.000000	0.000000	0.000000
3 vs. 6	0.0778	0.3503	0.000000	0.7895	0.5771	0.0901	0.000000	0.000000	0.0001
3 vs. 12	0.0741	0.3563	0.000000	0.7444	0.0147	0.6660	0.000000	0.000000	0.000000
6 vs. 12	0.2650	0.1012	0.3606	0.5890	0.0565	0.3272	0.0061	0.000000	0.0031

C2:0—acetic acid; C3:0—propionic acid; C4i—isobutyric acid; C4n—butyric acid; C5i—isovaleric acid; C5n—valeric acid; C6:0—caproic acid; C7:0—heptanoic acid.

**Table 2:** Correlations of SCFA concentrations at 1, 3, 6, and 12 months of age with gastrointestinal symptoms.

**1 Month**	**C2:0**	**C3:0**	**C4i**	**C4n**	**C5i**	**C5n**	**3,4-Methylovaleric**	**C6:0**	**C7:0**
Mucus in stool	−0.019	0.056	0.179	−0.147	−0.038	−0.087	−0.065	0.126	−0.087
Bloating/gas	−0.110	−0.107	−0.134	0.391	0.343	0.310	0.307	−0.337	0.305
Intestinal colic	0.216	−0.076	−0.163	0.321	−0.022	−0.113	−0.112	−0.311	−0.147
Regurgitation	−0.254	0.180	0.240	0.116	0.206	0.120	0.130	0.032	0.095
Green stools	−0.132	0.030	0.284	−0.184	0.048	0.006	0.058	0.244	0.040
Loose stools	−0.051	0.055	0.052	−0.222	0.079	−0.079	−0.012	0.197	−0.05
Constipation	0.042	0.139	−0.023	−0.138	0.245	−0.090	−0.070	−0.008	−0.110
**3 Months**	**C2:0**	**C3:0**	**C4i**	**C4n**	**C5i**	**C5n**	**3,4-Methylovaleric**	**C6:0**	**C7:0**
Mucus in stool	0.321	−0.316	−0.198	−0.317	−0.228	−0.134	0.435	−0.216	0.264
Bloating/gas	−0.234	0.011	−0.167	0.344	0.114	0.449	0.090	−0.088	0.277
Intestinal colic	0.195	−0.134	0.048	−0.212	−0.195	−0.133	−0.054	0.152	−0.048
Regurgitation	0.150	0.014	−0.341	−0.091	−0.140	0.009	0.053	−0.190	0.006
Green stools	−0.015	0.249	−0.143	0.014	0.05	−0.136	−0.070	−0.289	−0.188
Loose stools	0.141	−0.006	−0.040	0.084	−0.167	−0.148	−0.236	−0.174	−0.261
Constipation	0.115	0.144	−0.146	−0.136	−0.195	−0.148	−0.118	−0.083	−0.097
**6 Months**	**C2:0**	**C3:0**	**C4i**	**C4n**	**C5i**	**C5n**	**3,4-Methylovaleric**	**C6:0**	**C7:0**
Mucus in stool	0.056	0.040	−0.091	−0.178	0.226	0.174	0.115	−0.087	0.290
Bloating/gas	−0.284	−0.208	0.277	0.439	0.007	0.365	0.376	−0.049	0.148
Intestinal colic	0.082	−0.267	0.309	0.151	−0.266	−0.182	−0.054	0.040	−0.081
Regurgitation	−0.198	0.215	0.140	0.004	0.128	0.198	0.153	0.058	0.213
Green stools	0.188	−0.343	0.071	0.077	−0.274	−0.080	0.134	−0.030	−0.000
Loose stools	0.136	−0.003	0.204	−0.099	−0.219	−0.159	−0.029	−0.074	−0.198
Constipation	−0.398	−0.021	0.333	0.359	0.217	0.257	0.390	−0.008	0.344
**12 Months**	**C2:0**	**C3:0**	**C4i**	**C4n**	**C5i**	**C5n**	**3,4-Methylovaleric**	**C6:0**	**C7:0**
Mucus in stool	0.418	−0.535	−0.372	0.485	−0.253	−0.240	−0.606	−0.356	0.337
Bloating/gas	−0.535	0.516	0.435	−0.253	0.393	0.039	0.396	−0.133	−0.310
Intestinal colic	0.076	−0.170	0.099	0.010	0.204	−0.260	0.233	−0.071	−0.152
Regurgitation	0.020	0.173	0.148	−0.478	0.131	−0.230	0.397	0.053	0.433
Green stools	0.029	−0.103	−0.236	0.275	−0.059	−0.060	−0.004	−0.230	−0.380
Loose stools	−0.136	−0.180	0.228	0.348	0.336	−0.362	−0.018	−0.175	0.026
Constipation	−0.359	0.148	0.551	−0.112	0.580	−0.163	0.323	0.061	0.079

C2:0—acetic acid; C3:0—propionic acid; C4i—isobutyric acid; C4n—butyric acid; C5i—isovaleric acid; C5n—valeric acid; C6:0—caproic acid; C7:0—heptanoic acid.

**Table 3:** Body mass of infants.

Indicators	Mean	Min–Max	Median	SD
Newborn body mass [kg]	3.286	2.090–4.390	3.285	0.395
Body mass at 3 months of age [kg]	6.341	4.740–8.120	6.425	0.651
Body mass at 6 months of age [kg]	8.020	6.043–10.56	8.010	0.89
Body mass at 12 months of age [kg]	10.567	8.530–13.43	10.53	1.04

## Data Availability

The data are available upon request.
